# Association between self-control and health risk behaviors: a cross-sectional study with 9th grade adolescents in São Paulo

**DOI:** 10.1186/s12889-021-11718-4

**Published:** 2021-09-19

**Authors:** Roberta Corradi Astolfi, Maria Alvim Leite, Cassio Henrique Gomide Papa, Marcelo Ryngelblum, Manuel Eisner, Maria Fernanda Tourinho Peres

**Affiliations:** 1 Departamento de Medicina Preventiva, Faculdade de Medicina FMUSP, Universidade de Sao Paulo, Sao Paulo, SP, BR. Address: Av. Dr. Arnaldo, 455 - 2o. andar - sala 2214, São Paulo, Postal Code: 01246-903 Brazil; 2 Institute of Criminology, University of Cambridge and Professor of Developmental Criminology, Jacobs Center for Productive Youth Development, University of Zurich, Zurich, Switzerland

**Keywords:** Self-control, Multiple health risk behaviour, Adolescents, Low- and middle-income country

## Abstract

**Background:**

Self-control (SC) has been consistently found associated with diverse health risk behaviors (HRBs), but little research refers to low- and middle-income countries. Furthermore, there is evidence that some HRBs tend to aggregate, however studies with the specific purpose of addressing the relation between SC and multiple health risk behaviors (MHRBs) are rare. The objective of this study is to analyze these associations and provide evidence to help filling these gaps.

**Methods:**

A sample of 2106 9th grade students from the city of São Paulo responded a self-administered questionnaire in 2017. We tested the association of SC measured as an ordinal variable with four levels (higher, high, medium and low) with six HRBs (binge drinking, marijuana use, smoking, high consumption of ultra-processed food, sedentary behavior and bullying perpetration), in both separated and aggregated forms (MHRBs), controlling for potential confounders. Binary logistic regression was used to test the association between exposure (SC) and single outcomes. In order to analyze the association of SC with MHRBs, multinomial logistic regression was employed.

**Results:**

SC was associated with five of six HRBs investigated and with MHRBs. The effect size of the association of SC and MHRBs increased in a steep pattern with accumulation of more HRBs.

**Conclusion:**

Low self-control is associated with most HRBs investigated and the magnitude of the association increases when more than two or three HRBs are accumulated. There seems to be a group of adolescents in a position of pronounced vulnerability for MHRBs. This should be considered when designing public policy and prevention programs. In contexts of limited or scarce resources and public funds, interventions focusing the most vulnerable groups, instead of universal interventions, should be considered.

**Supplementary Information:**

The online version contains supplementary material available at 10.1186/s12889-021-11718-4.

## Background

It has long been recognized that much of the burden of health problems can be attributed to behavioral risk factors [1–3]. Common risk behaviors for several Noncommunicable Diseases (NCD) include tobacco and alcohol use, physical inactivity, as well as unhealthy diet [4]. Many of these behaviors tend to begin during the adolescence and persist throughout life [5]. Risk behaviors “can be defined as those that are potentially capable of threatening physical or mental health, in both present and future” [6].

Studies available in the literature indicate that different HRBs tend to co-occur and form clusters [7–9]. Among Brazilian adolescents, evidence suggests that the accumulation of two or more HRBs increases with age [10–12] and is more frequent among children attending public schools [12]. Despite some considerable accumulation of research [8], to our knowledge, studies on the association between socioemotional factors and clusters of HRBs are scarce and mainly concentrated in high income countries.

One important socioemotional factor is self-control, “the ability to self-regulate behavior and inhibit impulses” [13] or “the ability to pursue overarching goals despite short-term temptations, distractions or aversive states” [14].

Low self-control has been found to be associated with many negative outcomes [15]. In particular, among adolescents around the globe, low SC has been found to be positively associated with criminal offending, aggressive or delinquent behavior [13, 16–25], use of legal and illegal psychoactive substances [26–29], bullying perpetration [30, 31] and negatively associated with health promoting behaviors [32]. Similar associations have been found regarding dietary habits [20, 33] and sedentary behavior [34, 35]. Some longitudinal studies demonstrated that low SC during childhood is associated with negative outcomes in adulthood, with impairments in health, wealth and social adjustment, such as greater involvement in criminal activities [15, 36]. Studies with different research designs, samples and measurement instruments, show evidence of the association between SC and specific HRBs or a restricted set of similar behaviors, but few tested different HRBs simultaneously; exceptions worth noting are Moffit et al. [15] and de Winter et al. [37]. Furthermore, the relation of the SC and MHRBs has received scarce attention [37].

### The current study

The present study aims to investigate the association between self-control and six different HRBs (binge drinking, marijuana use, smoking, high consumption of ultra-processed food, sedentary behavior, and bullying perpetration), in both individual and aggregated forms (MHRBs), among adolescents in São Paulo, Brazil.

There is little evidence of the association of SC and a wide set of HRBs coming from the same study and evidence for the effect of SC on the accumulation of HRBs among adolescents is scarce [37]. There are important reasons for which these gaps should be filled. Additionally, to our knowledge, no study considered the effect of SC on HRBs in the Brazilian context.^1^

A better understanding of the association of SC with MHRBs can be useful for the development of prevention programs. Due to an alleged gradient effect of SC across the entire population, it has been argued that universal programs are the most interesting option from a public policy point of view [15]. Interventions based on opt-out schemes modify environmental conditions to induce the adoption of healthy behaviors as the default option [15]. An example is the prohibition of cigarette consumption indoors or in public buildings, making the decision to smoke more costly and thus inducing individuals to choose not to smoke. This type of policy does not intend to change individual traits, but only behavior and is applicable to the society at large.

Alternatively, to opt-out schemes, there are interventions aiming at the development of social skills. This model is individually based and believed to work best when applied early in childhood (or even during pregnancy). The Nurse-Family Partnership is the best evaluated program of early childhood development and it is based on intensive services to high-risk families and delivered by highly skilled professionals [38]. Comparatively, social skill interventions targeting middle childhood or adolescence, have shown more modest results [39]. A recent meta-analysis of randomized trials found consistent positive results for universal interventions (physical activity, mindfulness and yoga, family-based and other social and personal skills interventions) to improve self-regulation of children and adolescents [40]. However, greater effect-size was found among individuals considered at risk at baseline [40]. When it comes to the design of interventions aiming at the development of social skills, three major factors must be addressed: who should be invited (target versus universal approaches), when to intervene and the profile of personnel [38].

It does not seem that any of these models are mutually exclusive, but can be adopted in different forms and combinations in each particular context. In contexts with high rates of serious violence and social inequality [41], intensive and target programs for adolescents at risk for MHRB should be considered.

Also, if SC is related to such a wide variety of HRBs, it could replace programs targeting specific behaviors (such as abusive substance use). To sum up, understanding how levels of SC affect the occurrence of MHRBs is essential to guide public policy design.

The first hypothesis of the present study is that SC is associated with all six HRBs investigated. Consequently, an association of SC with the occurrence of MHRBs should also be expected. The second hypothesis is that the magnitude of the association of SC and MHRBs should increase manifold after more than two or three HRBs are accumulated.

## Methods

Data was collected in the research “São Paulo Project for the social development of children and adolescents” (SP-PROSO). SP-PROSO is the result of a partnership between the Department of Preventive Medicine of the University of São Paulo Medical School and the Violence Research Centre of the University of Cambridge.

The target population of SP-PROSO was composed of adolescents enrolled in the 9th grade in the city of São Paulo in 2017. The sample was stratified according to school type: state public, municipal public and private, and by conglomerate, taking each class as a draw unit. 156 classes, one class from each school, were randomly selected and 119 agreed to participate in the study. The minimum sample size in São Paulo was determined as 2849 students to allow estimates as low as 15% with a precision of 0.06 and deff = 1.7. Any adolescent present in classroom on the day of data collection, whose parents had not proscribed their child’s participation and who did not present any serious impairment that could prevent them from understanding or answering the questionnaire anonymously, was considered eligible. Considering the 2816 students present on the day of the survey; 96 refused to participate and 18 were excluded due to failed questionnaire completion, which resulted in a sample of 2702 adolescents, 94.5% of the estimated sample. A total of 2680 adolescents answered more than 80% of the questionnaire and were included in the data analysis. For this study, only questionnaires with all answers filled in the variables of interest were included, yielding a sample of 2106 adolescents.^2^

Students, individually, filled the printed questionnaires, in the presence of trained researchers and without the presence of teachers or other school professionals. All the questionnaires were reviewed upon completion to detect inconsistencies and missing values when students were asked to complete the section. All the questionnaires were anonymous for students and schools.

For the most part, SP-PROSO instrument is the same as the 6th wave of the “Zurich Project on the Social Development of Children” (Z-PROSO) [26] and the “*Proyecto Montevideo para el desarrollo social de niños y adolescents”* (M- PROSO) [42]. Additional questions were included to account for specific interests regarding the Brazilian context. All the questions used in this study are available in the Supplementary File. The translation process followed recommendations for culturally sensitive translation [43, 44].

### Outcome variables

Binge drinking: Adolescents were asked if they had taken five or more drinks in a single occasion over the 30 days prior to the survey. Possible responses were yes and no.

Smoking tobacco and marijuana use: Adolescents were asked about the frequency of use in the previous 12 months. Possible answers were “never”, “once”, “2 to 5 times”, “every month”, “every week” and “(almost) daily”. Responses were recorded as no (never) or yes (all other answers) as a binary variable.

High consumption of ultra-processed foods: Adolescents were asked how often they ate five types of foods in a regular week: sausages, crackers, packet snacks, treats (sweets, candies, chocolates) and sugary drinks. The possible answers ranged from never to 7 days. A score was calculated by the sum of answers. The sample was then divided into quartiles and the upper quartile was labelled as high consumption of ultra-processed foods.

Sedentary habits: Individuals were asked how much time each day they sat watching television, using the computer, talking to friends or doing other things (apart from time at school). Possible answers ranged from “less than one hour a day” to “more than eight hours a day”. Those who answered more than eight hours a day were considered sedentary.

Bullying perpetration: Following Alsaker [45, 46], five questions were asked about the repeated practice in the 12 months prior to the survey verifying: the practice of exclusion/ostracism; make fun/offend; hit/kick/pull hair; destroy/steal/hide belongings and sexually harassing. Bullying was considered to have occurred when the respondent reported at least one of those behaviours, in a frequency of at least once a month in the previous year.

Multiple health risk behaviors: A score was made summing the type of behaviors declared by each respondent. Scores of 4, 5 and 6 were combined because of the low number of cases in each score, so that the final variable ranges from 0 (no health risk behavior) to 4 (4 or more health risk behaviors).

### Exposure variable: self-control

Self-control was measured through a 10-item scale adapted from the self-control measure proposed by Grasmick et al. [47]. This reduced scale was used at the 6th wave of the longitudinal Z-PROSO project [48]. Respondents were presented with statements (i.e: “I often act on the spur of the moment without stopping to think”, “Sometimes I will take a risk just for the fun of it” and “If things I do upset people, it’s their problem, not mine”) and asked to choose from a Likert scale, ranging from 1 “totally disagree” to 4 “totally agree”. A score was calculated from the mean value of all answers for each participant (Cronbach’s alpha = 0.75; CFA: Loadings: 0.25–1.13; chi2 = 267.969 (31df); *p* < 0.001; RMSEA = 0.05; CFI: 0.95). The sample was then divided in quartiles named: highest, high, medium and low.

### Adjustment variables

Sociodemographic variables: Gender (0-female/1-male) and age (in years) were investigated. The variable Socioeconomic Status of the Family (SES) was created based on the questions from the School-Based Health Survey (PeNSE) in Brazil [49]. A score was calculated with seven items: ownership of consumer goods in the household (landline and car), personal belongings (cellphone, computer with internet and television), number of bathrooms in the house and having a monthly paid house keeper working at home. A summative weighted score (frequent items were given lower weights while most infrequent were given higher weights) was calculated as proposed by Levy et al. [50].

Important variables regularly associated with HRBs are moral values, attitude and beliefs towards violence [22, 24, 25], parenting techniques [22, 23, 31], peer delinquency [13, 18, 22, 28, 31, 51], school disorder [31], and violence/disorder in the neighborhood [13, 22]. Such variables were included in the analysis in order to isolate the possible independent association of SC with HRBs.

For the moral values scale, students were presented with a list with seven different actions someone his/her age could do and asked to evaluate in a scale ranging from “not bad at all” [1] to “very bad” [7]. Situations included lying to parents, teachers and other adults, playing truant at school, hitting someone for being insulted, stealing something worth about 5$ and insulting another adolescent for not liking him/her. Comparing to Z-PROSO, the SP-PROSO questionnaire presents two additional questions concerning the use of a gun to assault or rob. A mean score was calculated for the seven questions (Cronbach’s alpha = 0.83; CFA: Loadings: 1.28–1.82; chi2 = 88.834 (10df); *p* < 0.001; RMSEA = 0.05; CFI: 0.99).

Perceptions about positive parenting: Three questions (whether parents recognized and appreciated the adolescents’ efforts or achievements) that compose the positive parenting sub-dimension from the Alabama Parenting Questionnaire [52] were employed. Possible answers ranged from “never” to “often” in a four-point Likert scale. A mean score was calculated (Cronbach’s alpha = 0.82; CFA: Loadings: 0.41–1.62; chi2 = 341.843 (41df); *p* < 0.001; RMSEA = 0.05; CFI: 0.96).

Delinquent peer group: membership to a delinquent/transgressive peer group was assessed with two questions from the Eurogang Survey [53]. Initially, it was asked whether they considered themselves part of a group of friends. For those who had a positive answer, they were asked if any of their friends in the group took part in a list of nine actions, such as selling illegal drugs, getting protection money and carrying weapons. If the respondent answered that at least one member of the group has done at least one of the nine items, it was considered that he/she was part of a delinquent peer group.

School administrative status refer to whether the institution is private or public. Public schools include municipal and states government administration, but here both were included into one single category.

Exposure to school violence and disorder is a mean score containing 12 items assessing the frequency of witnessing or hearing about school violence, or school disorder, within the previous 12 months of the survey created by SP-PROSO team. Items included physical fights, drug selling, gun possessions and others. Answers were in a 4-point Likert scale from “never” to “often (5 or more times) (Cronbach’s alpha = 0.83; CFA: Loadings: 0.20–1.31; chi2 = 295.979 (35df); *p* < 0.001; RMSEA = 0.05; CFI: 0.97).

The scale of exposure to community violence was adapted from Children’s Exposure to Community Violence scale [54]. Students were asked if they had heard about or seen violent events that had occurred in the previous 12 months in the neighbourhood where they lived. Events included shots or shooting, arrests by the police, drug selling, assassinations, people carrying firearms in the street (other than the police or those authorised to use firearms), robbery and police bribery, in a total of 14 items (Cronbach’s α: 0.92). Possible answers ranged from never to often (five or more times) and a mean for the 14 questions was computed. (Cronbach’s alpha = 0.92; CFA: Loadings: 0.80–1.34; chi2 = 505.675 (59df); *p* < 0.001; RMSEA = 0.05; CFI: 0.98).

Table 1 summarizes the variables used in the analysis.

**Table 1:** Description of variables

	Variables	Details	Values
Outcome variables	Binge drinking	Had 5 or more doses in the same occasion in the previous month.	1 = yes, 0 = no
	Smoking	At least once in the previous year.	1 = yes, 0 = no
	Marijuana use	At least once in the previous year.	1 = yes, 0 = no
	High consumption of ultra-processed foods (UPF)	A score made by the sum of the scores by the answers to the following question: “How many days did you eat each of these food types on the past seven days? Answers ranged from 0 = none to 7 = every day. The sample score was divided into quartiles and the upper quartile was labelled as high consumption of ultra-processed foods (UPF).	1 = yes, 0 = no
	Sedentary habits	Do children spend more than eight hours seated apart from school hours?	1 = yes, 0 = no
	Bullying perpetration	Has committed with frequency of at least once a month in the previous year.	1 = yes, 0 = no
	Multiple health risk behaviors	A score was made summing the type of behaviours declared by each respondent. There are six health risk behaviours so that the possible values range from 0 to 6. Scores of 4, 5 and 6 were then combined as “4 or more”.	0, 1, 2, 3, 4 or more
Explanatory variable	Self-control	Behavioural scale assessing aspects of impulsivity, immediacy, risk and adventure seeking and self-centeredness. Answers ranged from “totally agree” to “totally disagree” and the mean was calculated for the sample. The sample was then, divided into quartiles. Highest scores are equivalent to lower self-control (more low self-control).	Highest, high, medium and low
Adjustment variables	Gender		1 = male, 0 = female
	School administrative status		1 = public, 0 = private
	Delinquent peer group		1 = yes, 0 = no
	Age	Discrete (completed years)	
	Socioeconomic status	A score was calculated with seven items: possession of consumer goods in the household (landline, computer, car, etc.) and having a monthly paid house cleaner working at home. Most frequent items were given lower weights while most infrequent were given higher weights.	
	Positive parenting	A mean score was calculated from three questions about recognition for efforts/accomplishments (ex.: “your parents let you know when you have done a good job with something”). Each question ranged from never (1) to frequently (4).	Possible values range from 1 to 4
	Morality index	A mean score was calculated from seven questions about how bad the person thought it was to do certain things (ex.: “lie to his/her parents, teachers or other adults”). Each question ranged from not bad at all (1) to very bad (7).	Possible values range from 1 to 7
	Exposure to school violence and disorder	A mean score of 12 items assessing the prevalence of witnessing or hearing about school violence or school disorder. Answers were in a 4-point Likert scale from “never” to “often (5 or more times)”.	Possible values range from 1 to 4
	Exposure to community violence	A mean score was calculated considering the frequency of occurrence of situations such as drug selling, robbery and police bribery, in the previous 12 months. Each question ranged from not never (1) frequently (4).	Possible values range from 1 to 4

### Statistical analysis

Analyses were performed using the software Stata 15.1 All the analyses considered sampling weights and were run using the *svy* module. A descriptive analysis with the calculation of proportions for categorical variables and means for continuous and discrete variables, followed by 95% Confidence Intervals was conducted.

To test the association between exposure (self-control) and single outcomes (binge drinking, marijuana use, smoking, high consumption of ultra-processed food, sedentary behavior, and bullying perpetration), binary logistic regression, adjusting for covariates, has been used. To analyze the association of SC with the accumulation of HRBs, the multinomial logistic regression has been employed.

### Robustness checks

We chose to categorize SC according to quartiles to calculate specific OR according to the capacity to exercise self-control. However, this approach has some disadvantages, such as the arbitrary definition of a cut-off point for the mean SC categories. To check for the consistency of our results, we re-estimated the adjusted model having SC as a continuous variable. This model results in a single OR for the SC variable that was expected to be positive (OR > 1), in the case, lowering the capacity of self-control showed to be associated with a higher odds of health risk behaviors. The result is reported in Tables 6 and 7 in the appendix. The results remained the same: each unity increase in low self-control score (meaning lowering the self-control capacity) is associated with higher odds for all HRBs.

## Results

### Sample descriptions

As shown in Tables 2, 50.6% of respondents in our sample were boys and the mean age was 14.8 years. Socioeconomic status ranged from 0 to 13.56 and its mean was 7, whereas 33% of adolescents attended private schools. The mean score for SC for the whole sample was 2.2 (CI 95% 2.2–2.3).^3^ Mean score for each quartile of SC and the descriptive statistics of the adjusting variables are displayed in Table 2.

**Table 2:** Characteristics of the sample – control and explanatory variables - consisting in 9th grade students from elementary schools in São Paulo, Brazil, 2017 (SP-Proso study; n = 2106)

Variables	Categories	Frequency yes (=1) % (95% CI)	Mean; (CI 95%)
Gender (girls = 0)		50.55(48.42–52.68)	–
Age		–	14.80 (14.77–14.84)
Socioeconomic status		–	7.00(6.76–7.24)
School status (public = 0)		32.99 (30.36–35.74)	–
Self-control	Highest	26.22 (24.23–28.32)	1.63 (1.60–1.66)
	High	23.89 (21.82–26.10)	2.10 (2.09–2.11)
	Medium	25.08 (23.31–26.95)	2.40 (2.40–2.41)
	Low	24.80 (22.72–27.01)	2.84 (2.81–2.86)
Delinquent peer group (No = 0) |		11.66(9.63 14.04)	–
Positive parenting		–	3.04 (3.01–3.07)
Moral values		–	5.24 (5.16–5.32)
Exposure to school violence and disorder		–	1.79 (1.76–1.83)
Exposure to community violence		–	1.75 (1.71–1.78)

Table 3 presents the description of the sample concerning HRBs. The most common HRBs referred in the sample were binge drinking (28.1%) and high consumption of ultra-processed food (22.9%) while the least common was marijuana use (10.8%). Concerning MHRBs, 42.4% of respondents referred none of the six HRBs of interest. For 28.3%, one of the behaviors was present, and for 5.8%, four or more behaviors were reported.

**Table 3:** Characteristics of the sample – outcome variables - consisting in 9th grade students from elementary schools in São Paulo, Brazil, 2017 (SP-Proso study; n = 2106)

Variables	Categories	Frequency yes (=1) % (95% CI)	Mean; (CI 95%)
Binge drinking		28.14 (25.80–30.60)	–
Smoking		17.81 (15.83–19.98)	–
Marijuana use		10.76 (8.87–12.99)	–
High consumption of UPF		22.89 (20.82–25.09)	31.93 [high](31.69–32.17) 15.96 [others](15.56–16.36)
Sedentary habits		14.77 (12.87–16.90)	–
Bullying perpetration		14.69 (12.80–16.82)	–
Multiple HRBs	None	42.44 (39.98–44.94)	–
	One	28.25 (26.07–30.53)	–
	Two	14.68 (13.01–16.54)	–
	Three	8.80 (7.47–10.43)	–
	Four or more	5.79 (4.62–7.24)	–

### Bivariate and multivariate analysis

Table 4 presents crude and adjusted odds ratios (OR) for the association between SC and each HRB investigated. SC is associated with all outcomes in the bivariate analysis (crude OR). For binge drinking and smoking, any decrease in SC, as compared to the highest level, was associated with higher odds of the outcome with statistical significance. For marijuana, high consumption of ultra-processed food and bullying perpetration, a negative and significant association was found only for those in the medium and low SC groups as compared to those with highest SC capacity. For sedentary habits, a negative association was found only for those in the low SC group.

**Table 4:** Raw and adjusted association between self-control and HRBs among 9th grade elementary students. São Paulo, Brazil (SP-Proso study). *N* = 2106. São Paulo, 2017. OR adjusted (CI 95%)

Outcomes	Level of self-control	OR (CI 95%)	
		Crude	Adjusted
Binge drinking	Highest	1	1
	High	1.47* (1.00–2.14)	1.39 (0.93–2.07)
	Medium	3.05** (2.14–4.34)	2.45** (1.71–3.52)
	Low	7.20** (5.02–10.33)	4.42** (3.05–6.40)
Smoking	Highest	1	1
	High	1.87 * (1.09–3.23)	1.82*(1.03–3.23)
	Medium	3.28** (1.99–5.41)	2.58** (1.48–4.49)
	Low	6.73** (3.95–11.48)	3.87** (2.22–6.74)
Marijuana use	Highest	1	1
	High	1.02 (0.57–1.80)	0.95 (0.52–1.73)
	Medium	2.20** (1.45–3.34)	1.50 (0.95–2.38)
	Low	4.01** (3.27–7.67)	2.42** (1.52–3.85)
High consumption of UPF	Highest	1	1
	High	1.10 (0.76–1.59)	1.07 (0.73–1.57)
	Medium	1.67** (1.15–2.43)	1.44 (0.99–2.10)
	Low	2.94** (2.14–4.04)	2.33** (1.61–3.38)
Sedentary habits	Highest	1	1
	High	1.10 (0.72–1.70)	1.02 (0.65–1.61)
	Medium	1.22 (0.79–1.90)	1.09 (0.69–1.70)
	Low	1.78** (1.17–2.70)	1.41 (0.88–2.26)
Bullying perpetration	Highest	1	1
	High	1.55 (0.97–2.45)	1.38 (0.84–2.27)
	Medium	2.44** (1.52–3.94)	1.86*(1.14–3.03)
	Low	4.26** (2.81–6.48)	2.64** (1.71–4.07)

*p < 0.05 **p < 0.01. Adjusted for gender, age, socioeconomic status, school status, uent peer group, positive parenting, moral values, exposure to school violence and disorder and exposure to community violence

When control variables were added to each model, the general tendency for an increase in the odds ratio persisted. SC is associated with all HRBs except for sedentary habits. For high and medium SC groups, the strongest associations were with smoking (OR = 1.8 and 2.6, respectively). In the low SC group, the strongest association was with binge drinking. Bullying perpetration presented a pattern similar to binge drinking, since only associations of SC in the medium and low groups were significant. The association of marijuana use and high consumption of ultra-processed food and SC were significant only for the lower SC group and the respective OR were 2.4 and 2.3.

Regarding the sedentary behaviour, the association with SC lost its significance in the adjusted model for São Paulo adolescents, despite being positive. According to Gottfredson & Hirschi [55], the preference for physical activity over contemplative activity is one of the characteristics of people with low-self control. Following this, the scale proposed by Grasmick et al. [47] includes one dimension that reflects this preference as does the adapted version used in this study. This could explain our discrepant result: once preference for physical activity is common to those with low SC, they would tend to be less sedentary. To deal with this possibility, we ran logistic regressions again (Appendix, Table 8) without the two items that composes the physical activity dimension in the SC scale and an OR of 1.6 (IC95% 1.03–2.55) appeared with significance for the low SC group.

Table 5 presents the association between self-control and MHRBs. It is possible to note an increase in the OR across self-control categories from the highest to the low group for one, two, three and four or more HRBs. High SC was not associated with any amount of risky behavior. Low SC, in its turn, was associated with every number of MHRBs, and the more behaviors were included, the stronger was the association. Medium level of SC was associated with two or more health risk behaviors, and the association was stronger in groups of respondents who concentrated four risk behaviors or more.

**Table 5:** Association (adjusted only) between self-control and MHRBs among 9th grade elementary students. São Paulo, Brazil (SP-Proso study). N = 2106. São Paulo, 2017

Level of self-control	MHRBs Adjusted OR (CI 95%)			
	One	Two	Three	Four or more
Highest***	*1*	*1*	*1*	*1*
High	0.99 (0.70–1.41)	1.85 (0.99–3.45)	1.20 (0.60–2.38)	1.58 (0.56–4.46)
Medium	1.26 (0.91–1.76)	3.00**(1.74–5.15)	2.91** (1.57–5.40)	4.02** (1.53–10.55)
Low	1.84** (1.19–2.84)	5.94**(3.32–10.63)	7.13** (3.82–13.30)	15.19** (5.99–38.53)

*p < 0.05 **p < 0.01. Adjusted for gender, age, socioeconomic status, school status, delinquent peer group, positive parenting, moral values, exposure to school violence and disorder and exposure to community violence. ***Reference Category

Being in high SC capacity group does not increase the odds of presenting one HRB as compared to the highest SC group (OR: 1, CI 95% 0.7–1.4). For the medium SC capacity group, associations are positive for any number of HRBs, but not significant for presenting one HRB. For the low SC capacity group, the association was positive and significant for one, two, three and four or more HRBs, with OR expressing a stronger magnitude for the association. As compared to the reference category, the odds of presenting one HRB in this group was 1.8, 5.9 for two HRBs, 7.1 for three HRBs and 15.2 for four or more HRBs.

## Discussion

In the past 30 years, self-control has been found related to a wide set of undesirable outcomes, across different disciplines and population samples [15]. In the present study, we investigated the association of SC with six different HRBs (binge drinking, marijuana use, smoking, ultra-processed food consumption, bullying perpetration and sedentary behavior), separately and as an aggregated measure of MHRBs, in a sample composed of adolescents in the city of São Paulo.

In our study, adolescents belonging to the low SC group presented higher odds of engagement in binge drinking, smoking tobacco, marijuana use, bullying perpetration and of presenting a pattern of high consumption of ultra-processed food, when compared to adolescents in the reference group (highest SC group).

Our findings are in accordance with those in international literature. The use of legal and illegal psychoactive substances has been found to be associated with low SC among adolescents in western high-income countries [26, 27, 29] and east Asia [28], bullying perpetration was found associated with low SC in a sample of Hispanic adolescents in a US city [31] and Macanese teenagers [30]. Concerning ultra-processed food, our results point in a similar direction to the findings for Australian boys and girls among whom negative control was associated with binge eating [34] and for American adolescents, among whom poor self-control was positively associated with saturated fat intake and good self-control with fruit, vegetable, and fiber intake [33].

The positive association of low SC and sedentary habits lost significance in the adjusted model and was found significant with an adapted scale that did not have the “preference for physical activities” dimension. Xiang et al. [35] found a positive association of self-control and sedentary behavior. It is also interesting to compare our result with Wills et al. (2007) who observed that good self-control was found to be negatively associated with sedentary behaviour, but poor self-control was not associated, either negatively or positively. Thus, the relationship of SC with sedentary behavior demands more in-depth studies, preferably with a sample including adults for whom the association might appear and with different forms of variable operationalization.

The hypothesis that SC would be associated with so many different human behaviors was present in the General Theory of Crime [55]. So far, the evidence of the association of SC and HRBs has mostly come in pieces, with each study testing specific constructs of SC and a few similar outcomes of interest, whereas only a handful of research addressed together HRBs that are very different from each other such as Moffit [15] and de Winter [37]. This study adds evidence that the association found between SC and so many different behaviors is not the product of scales tailor-made to present association with certain outcomes, but that this is a construct that captures the relation between the capacity to “delay gratification, control impulses, and modulate emotional expression” [15] and human behavior resulting from the conflict of desire and a higher-order goal [56].

While Moffit et al. [15] found a significant linear associations of SC and health, wealth, and crime, Mears et al. [21] found nonlinear two-threshold effect of SC on offending among US adolescents. Our study was not designed to account for the linearity of those associations, but our findings are more compatible with those presented by Mears et al. [21]. Based on the present results, it is not possible to ascertain that any amount of change in SC would produce a change in the frequency of outcomes of interest. For most of the outcomes investigated, except smoking, the increase in OR was significant only for those in the medium and low SC groups. In other words, our results point to the existence of a threshold, below which self-control begins to act as risk factor for HRBs in adolescence.

Our second hypothesis was that the association of SC with MHRBs would increase when new HRB were added. This hypothesis was also supported by the present results. So, even if future evidence supports the linear association hypothesis, since SC is related to many different outcomes, it is important to consider how the magnitude of the association changes when those different outcomes are considered together.

This specific discussion has been largely neglected by the literature, however such approach is of great importance because of its practical implications. Based on the finding that “health, wealth, and crime outcomes followed a gradient across the full distribution of self-control in the population”, Moffit et al. [15] suggest that universal type interventions to improve SC or mitigating its negative effects would be the most valuable, considering the net effect in prosperity and well-being of the population, with the advantage of not stigmatizing any groups and gaining widespread citizen support.

Our results do not challenge this position, but shed light to the complementary nature of different public policy designs. Public funds are always limited or scarce and interventions focusing the most vulnerable youths should be considered, specially taking in account that early child development programs are, at present, far from universal. In contexts with high rates of violence and inequality, the benefits of interventions focused on specific groups should be even higher, tackling a number of different undesirable outcomes. Working with a small portion of the population, even the most expensive programs would probably worth the investments. However, for solid conclusions, more evidence must be gathered and, for public policies, the population attributable risk must be estimated.

Another consideration should be made concerning adolescents in the other extreme, the upper intermediate level of SC. Jessor [1] states that the rationale underneath risk behaviors are not restricted to their outcomes, how ever adverse or undesirable they may be. Binge drinking, smoking and marijuana use can serve typical adolescent goals as peer acceptance and a sense of maturity and independence. Ultra-processed food consumption is pleasurable, and consumption related risk behaviors can be a way of dealing with anxiety. While the rationality of bullying perpetration can be more difficult to admit, given its consequences to others, and sedentary behavior can only be explained by immediate gratification, these two behaviours are not, necessarily, irrational or pathological. Improving SC of adolescents through universal interventions, as proposed by Moffit et al. [15] would, certainly minimize the frequency of negative behaviors and, consequently, their risks. However, if we consider the upper intermediate level of SC, improving this individual trait may not have such important gains as providing adolescents with alternative channels to achieve the same goals, since those behaviors might have been rationally chosen rather than just unintended consequence of immediate temptation.

Lastly, it is important to address the context in which the study was carried out. Since the Brazilian context is different from those where most part of other studies on SC were carried out (high-income and/or Asian countries), these results add relevant confirmatory evidence to the hypothesis that SC is a variable associated with a wide range of undesirable behaviors. The practical implications of these results, specifically concerning the adoption of combinations of universal and focused strategies, are of particular importance to other Latin-American countries that share similar social structures and patterns of violence and inequality and scarce public funds.

### Limitation of the study

Our results should be interpreted considering some limitations of the study. The SP-PROSO study is a school-based cross-sectional survey and for that matter, its first limitation is that it does not provide evidence on temporal sequence between exposure and outcomes. Longitudinal studies carried elsewhere provided evidence to the hypothesis that the causal path is from SC to HRBs [15, 19, 26]. A cohort study among a sample of adolescents in Brazil would be, nevertheless, a gain to our understanding of the phenomenon.

Secondly, the generalization of results is restricted, since the sample was representative of those present on the day of the data collection and that have not refused to participate in the survey. Furthermore, some of the selected schools refused to participate. Since information about either nonparticipant schools or absent adolescents is not available, it is not possible to estimate such bias in the study. Adolescents can be absent from school for a variety of reasons that could affect anyone regardless of their level of SC, from sickness, to feeling unsafe in the path to school. However, it is reasonable to suppose that students with lower SC are more prone to truancy and that they were overrepresented in the group of absent students, causing selection bias. In fact, in our sample, the correlation of the variable measuring truancy and SC is 0.23 and significant. However, it is also reasonable to suppose that truant students are also more likely to engage in most of the HRBs investigated here, such as binge drinking, smoking, use of marijuana and bullying perpetration. In that sense, we have no good reason to suppose that the association of exposure and outcome could be underestimated. On the other hand, we can expect that prevalence is underestimated, for both exposure and outcomes investigated.

Scales used in this study have been validated and used in multiple samples from different parts of the world, but they have not been fully validated in Brazil.

## Conclusion

The first hypothesis of the present study was that SC is associated with six HRBs investigated, which is largely supported by our results. Furthermore, the results fully supported the second hypothesis that the magnitude of the association of SC and MHRBs should increase once more than two or three HRBs accumulate.

We believe that the findings and discussions presented in this study make relevant contributions to the knowledge about SC and public policy design concerning the wellbeing and health of adolescents across the globe.

### Supplementary Information

**Additional file1.**

## Data Availability

The datasets used and/or analysed during the current study are available from the corresponding author on reasonable request.

## Appendix

**Table 6:** Association between self-control (continuous) and HRBs among 9th grade elementary students. São Paulo, Brazil (SP-Proso study). *N* = 2106. São Paulo, 2017. OR adjusted (CI 95%)

Outcomes	Ajusted OR (IC 95%)
Binge drinking	**3.68**** **(2.74–4.94)**
Smoking	**3.04**** **(2.02–4.56)**
Marijuana use	**2.11**** **(1.43–3.11)**
High consumption of UPF	**2.01**** **(1.50–2.69)**
Sedentary habits	1.18 (0.83–1.67)
*Bullying* perpetration	**2,54**** **(1.82–3.56)**

**p* < 0.05 ***p* < 0.01

Adjusted for gender, age, socioeconomic status, school status, transgressive peer group, positive parenting, moral values, school disorder and neighbourhood disorder

**Table 7:** Association (adjusted only) between self-control (continuous) and MHRBs among 9th grade elementary students. São Paulo, Brazil (SP-Proso study). N = 2106. São Paulo, 2017

	MHRBs Adjusted OR (CI 95%)			
	One	Two	Three	Four or more
Self-control	1.55** (1.13–2.12)	3.63** (2.45–5.38)	6.20** (3.80–10.13)	11.55** (6.09–21.90)

*p < 0.05 ***p* < 0.01

Adjusted for gender, age, socioeconomic status, school status, delinquent peer group, positive parenting, moral values, exposure to school disorder and exposure to neighbourhood disorder

**Table 8:** Association between self-control (without items of physical activity) and sedentary behaviour among 9th grade elementary students. São Paulo, Brazil (SP-Proso study)

Outcome	Level of self-control	OR (CI 95%)	
		Crude	Adjusted
Sedentary habits	Highest	1	1
	High	1.07 0.73–1.56	0.97 (0.66–1.43)
	Medium	1.48 1.00–2.20	1.30 (0.88–1.93)
	Low	1.98** 1.33–2.96	**1.62** ***** **(1.03–2.55)**

*N = 2105. São Paulo, 2017. OR crude and adjusted (CI 95%).*

*p < 0.05 **p < 0.01

Adjusted for gender, age, socioeconomic status, school status, delinquent peer group, positive parenting, moral values, exposure to school disorder and exposure to neighbourhood disorder.

## Abbreviations

SC: Self-control
HRB: health risk behaviors
MHRB: multiple health risk behavior
NCD: Noncommunicable diseases
SP-PROSO: São Paulo Project for the social development of children and adolescents
Z-PROSO: Zurich Project on Social Development of Children
M-PROSO: Montevideo project on Social Development of Children
PeNSE: School-Based Health Survey (Brazil)
UPF: Ultra-processed foods
OR: Odds ratio

## Footnotes

1 To our knowledge, only Rebellon [51] included subjects from Brazil, adolescents or otherwise, in a study of crime. However, it was a convenience sample of less than 400 college students.

2 For the scales constructed by mean of responses, a minimum of 80% response was adopted as criterion of inclusion. For summative scales, only questionnaires with all answers in the scale were included.

3 Not shown.
