# Supplementary material for: Association between self-control and health risk behaviors: a cross-sectional study with 9th grade adolescents in São Paulo
Source: BMC Public Health. 2021 Sep 19;21:1706. doi: 10.1186/s12889-021-11718-4 (PMC8451098; doi:10.1186/s12889-021-11718-4)
Supplement: Supplementary file 1 — Additional file1. [file 12889_2021_11718_MOESM1_ESM.docx]

PUBH-D-20-05934 – Supplementary material - SP-PROSO Instrument

Article title: Association between self-control and health risk behaviours: a cross-sectional study with 9th grade adolescents in São Paulo

For the most part, SP-PROSO instruments is the same as the 6th wave of the “Zurich Project on the Social Development of Children” (Z-PROSO) and the “Proyecto Montevideo para el desarrollo social de niños y adolescents” (M- PROSO). Additional questions or items were included to account for specific interests about the Brazilian context (as indicated by notes * and ^a^). The original questionnaire is 23 pages long and this translation contains only the questions used in the study “Association between self-control and health risk behaviours: a cross-sectional study with 9th grade adolescents in São Paulo”.

**General sociodemographic information**

| Let's start with some general questions about you and your family | Vamos começar com algumas perguntas gerais sobre você e sua família |
| --- | --- |
| Your date of birth ___ / ___ / | Sua data de nascimento ___/___/ |
| Gender: Male - Female | Sexo: Masculino – feminino |

*School status was coded by researchers

**Self-control scale**

| How you see yourself - Here are some more general questions.; First, they are about how you see yourself. Below are various statements. Please mark with a cross, how true these statements are for you.  false / more false than true / more true than false / true | Como você se vê - Agora voltamos para mais algumas perguntas gerais. Primeiro, vamos falar sobre como você se vê. Abaixo aparecem diferentes afirmações. Por favor, marque com um X o quanto você concorda ou discorda de cada uma delas.  Discordo totalmente / Discordo / Concordo / Concordo totalmente |
| --- | --- |
| I often act on the spur of the moment without stopping to think | Quase sempre faço coisas sem pensar. |
| I often do whatever brings me pleasure here and now, even at the cost of some distant goal. | Eu sempre faço o que quero sem pensar nas consequências a longo prazo. |
| Sometimes I will take a risk just for the fun of it. | Às vezes eu faço coisas perigosas e corro riscos somente por diversão. |
| Excitement and adventure are more important to me than security. | Para mim, emoção e aventura são mais importantes do que segurança. |
| I like to get out and do things more than I like to read or contemplate ideas | Eu prefiro sair e fazer algo fora de casa a ficar em casa lendo ou pensando. |
| If I had a choice, I would almost always rather do something physical than something mental. | Quando eu posso escolher, prefiro fazer atividades físicas a realizar atividades em que preciso ficar pensando. |
| I will try to get the things I want even when I know it's causing problems for other people. | Eu tento conseguir o que quero mesmo quando sei que isso causa problemas para outras pessoas |
| If things I do upset people, it's their problem not mine. | Eu não me importo quando os outros se chateiam por algo que eu fiz. |
| I lose my temper pretty easily. | Perco o controle muito rápido. |
| If I don’t get something I want immediately, I get pretty angry | Quando eu não consigo imediatamente o que quero, fico com raiva muito rapidamente |

**Bullying perpetration scale**

| This part is about bullying. Adolescents can be very mean to each other sometimes. And you? [...] In the last year, have you bullied other adolescents? This could be, for example, on the way to school, when being out, at home or even on the internet. How many times did you?  Never / 1 to 2 times / 3 to 10 times / about once a month / about once a week / (almost) every day | Agora vamos falar sobre abuso, bullying e maus tratos. Às vezes, adolescentes podem trata mal uns aos outros. Qual a sua experiência sobre isso? [...] E você? Você assediou ou maltratou outro adolescente neste último ano? Isso pode ter acontecido na escola, no caminho da escola, no seu tempo livre, em casa ou na internet. Quantas vezes, desde agosto de 2016 você...  Nunca / 1 ou 2 vezes / 3 a 10 vezes / Uma vez por mês / Uma vez por semana / Todo dia |
| --- | --- |
| …purposely ignored or excluded another youth? | Ignorou ou excluiu um outro adolescente de propósito? |
| …laughed at, mocked or insulted another youth? | Riu de outro adolescente, tirou sarro de outro adolescente ou ofendeu outro adolescente? |
| …hit, bitten or kicked another youth, or pulled their hair? | Bateu em outro adolescente, mordeu outro adolescente, chutou outro adolescente ou puxou o cabelo de outro adolescente? |
| …purposely stole, broken or hidden another youth's things? | Pegou, destruiu ou escondeu alguma coisa de outro adolescente de propósito? |
| …sexually harassed (eg: hit on, groped) another youth? | Assediou outro adolescente (por exemplo: deu em cima de forma invasiva que provocou desconforto, tocou outro adolescente ou falou algo constrangedor?) |

**Use of tobacco and marijuana**

| Listed below are some drugs, intoxicants and other substances. Have you ever taken this?  Yes / No | Abaixo estão alguns tipos de droga, cigarro e outras substâncias. Você já usou alguma delas?  Sim / Não |
| --- | --- |
| Cigarettes, tobacco, shisha | Tabaco, cigarro, charuto, cachimbo, etc. |
| Hash, "pot", cannabis, marijuana | Haxixe, Maconha |

**Binge drinking**

| Have you drunk alcohol in the last 30 days? If yes, on how many days did you drink 5 or more glasses of alcohol (eg.: beer, wine, vodka, mixed drins etc) in a row?  On ____ days | Você tomou bebidas alcoólicas nos últimos 30 dias? [Se sim] Em quantos dias você tomou 5 ou mais doses de bebida alcoólica (cerveja, vinho, vodka, bebidas misturadas, etc.)?  Em ____ dias |
| --- | --- |

**Consumption of ultra-processed food^a^**

| The next questions are about your diet. Take into account everything you've eaten at home, at school, on the street, in cafeterias, in restaurants or anywhere else in the past 7 days. IN THE LAST 7 DAYS, how many days did you eat or drink:  Didn't eat / 1 day / 2 days / 3 days / 4 days / 5 days / 6 days / Every day | As próximas perguntas referem-­‐se a sua alimentação. Leve em conta tudo o que você comeu em casa, na escola, na rua, em lanchonetes, em restaurantes ou em qualquer outro lugar nos últimos 7 dias. NOS ULTIMOS 7 DIAS, em quantos dias você comeu ou bebeu:  Não comi / 1 dia / 2 dias / 3 dias / 4 dias / 5 dias / 6 dias / Todos os dias |
| --- | --- |
| Fried snacks. Ex: Potato chips (not counting the packet potato) or fried snacks such as chicken drumstick, fried kibbeh, fried pastry, acarajé, etc.. | Salgados fritos. Ex: Batata frita (sem contar a batata de pacote) ou salgados fritos como coxinha de galinha, quibe frito, pastel frito, acarajé, etc. |
| Hamburger, bologna, salami, ham, nuggets or sausage. | Hambúrguer, salsicha, mortadela, salame, presunto, nuggets ou linguiça. |
| Cookies or crackers. | Biscoitos ou bolachas, doces ou salgados. |
| Packaged snacks or packaged potato chips. | Salgadinho de pacote ou batata frita de pacote. |
| Goodies (candies, candies, chocolates, chewing gum, chocolates or lollipops) | Guloseimas (doces, balas, chocolates, chicletes, bombons ou pirulitos) |
| Soda, powdered juice, soft drink or juice box. | Refrigerante, suco de pozinho, refresco ou suco de caixinha. |

**^a^** SP-Proso instrument

**Sedentary behavior^a^**

| On a typical weekday, how long are you sitting watching television, using a computer, playing video games, talking to friends or doing other activities while sitting? (Do not count Saturday, Sunday, holidays and sitting at school).  Less than 1 hour a day / 1 to 2 hours a day / 3 to 4 hours a day / 5 to 6 hours a day / 7 to 8 hours a day / More than 8 hours a day | Em um dia de semana comum, quanto tempo você fica sentado (a) assistindo televisão, usando computador, jogando videogame, conversando com amigos (as) ou fazendo outras atividades sentado (a)? (Não contar sábado, domingo, feriados e o tempo sentado na escola).  Menos de 1 hora por dia / 1 a duas horas por dia / 3 a 4 horas por dia / 5 a 6 horas por dia / 7 a 8 horas por dia / Mais de 8 horas por dia |
| --- | --- |

**Morality index**

| How do you see this? - Below are various things that adolescents can do. Please indicate whether you think it is not bad at all, not bad, bad or very bad when adolescents of your age do these things. How bad is it when someone your age…?  1 – 7 | O que você acha disso? Agora vamos falar de coisas que os adolescentes podem fazer. Por favor, considerando (1) nada grave e (7) muito grave, marque o quão grave você considera as atitudes abaixo. Quão grave você acha que é quando alguém da sua idade...?  1 a 7 |
| --- | --- |
| ...lies to his/her parents, teachers, or other adults? | ...mente para os pais, os professores ou outros adultos? |
| ...plays truant on purpose? | ...mata aula de propósito? |
| ...hit someone because he/she was insulted? | ...bate em uma pessoa e a machuca porque ela o ofendeu? |
| ...steals something worth less than 5 CHF? (about 5$) | ...rouba algo que vale menos de R$ 15,00? |
| ... attacks another person with a gun with intentions to seriously injure them? * | ...ataca outra pessoa com uma arma com intenções de feri-la seriamente? * |
| ... uses a gun to force or compel someone else to give you your money or things? * | ...usa uma arma para forçar ou obrigar outra pessoa a lhe dar seu dinheiro ou suas coisas? * |
| ...insults other adolescents because he/she doesn't like them? | ...ofende outros adolescentes dos quais não gosta? |

*Additional items of the Brazilian instrument.

**Exposure to school violence and disorder^a^**

| School - Thinking about the last 12 MONTHS, how often have you witnessed (seen) or heard that any of the situations below happened at the school where you currently study (this school)?  Never / Few times (1 or 2) / Sometimes (3 or 4) / Many times (5 or +) | Escola - Pensando nos últimos 12 MESES, com que frequência você presenciou (viu) ou ouviu falar que alguma das situações abaixo aconteceu na escola onde você estuda atualmente (esta escola)?  Nunca / Poucas vezes (1 ou 2) / Algumas vezes (3 ou 4) / Muitas vezes (5 ou +) |
| --- | --- |
| Fight with physical aggression among students | Briga com agressão física entre alunos |
| Fights with physical aggression involving students and teachers and / or employees | Brigas com agressão física envolvendo alunos e professores e/ou funcionários |
| Bullying among students (humiliation, bruising, verbal aggression, cursing, persistent and recurring against specific students) | Bullying entre alunos (humilhação, pirraçãs, agressão verbal, xingamentos, persistentes e recorrentes contra alunos específicos) |
| Sale and / or consumption of drugs at school | Venda e/ou consumo de drogas na escola |
| Students carrying firearms and / or knife | Alunos portando armas de fogo e/ou faca |
| Some type of sexual violence or sexual assault involving students | Algum tipo de violência ou agressão sexual envolvendo alunos |
| Some type of sexual violence or sexual assault involving students and teachers and / or staff | Algum tipo de violência ou agressão sexual envolvendo alunos e professores e/ou funcionários |
| Fights with verbal offense between students | Brigas com xingamentos entre alunos |
| Fights with verbal offense involving students and teachers and / or staff | Brigas com xingamentos envolvendo alunos e professores e/ou funcionários |
| Robberies or thefts occurred inside the school | Roubos ou furtos acontecidos no interior da escola |
| Vandalism by a student against school property (eg breaking, destroying, graffiti) | Vandalismo praticado por aluno contra os bens da escola (p.ex quebrar, destruir, pichar) |
| Vandalism by teachers and / or staff against school property (eg breaking, destroying, graffiti) | Vandalismo praticado por professores e/ou funcionários contra os bens da escola (p.ex quebrar, destruir, pichar) |

**Exposure to community violence and disorder^a^**

| Thinking about the last 12 months, how often have you heard that any of the following happened in the neighborhood where you live:  Never / once or twice / A few times / Many times | Pensando nos últimos 12 meses, com que frequência você ouviu falar que alguma das situações abaixo aconteceu no bairro onde você mora:  Nunca / Poucas vezes (1 ou 2) / Algumas vezes (3 ou 4) / Muitas vezes (5 ou +) |
| --- | --- |
| Guns being shot | Tiros ou tiroteios |
| Somebody arrested | Alguém foi preso pela polícia |
| Drug dealing | Alguém vendendo drogas |
| Someone beaten up | Alguém foi espancado ou agredido fisicamente |
| A murder | Alguém foi assassinado |
| A house broken into | Alguém teve a casa invadida/roubada |
| Someone stabbed | Alguém foi esfaqueado |
| Someone shot | Alguém levou um tiro |
| People walking with firearms on the street (other than police officers or people authorized to use firearms) | Pessoas circulando com armas de fogo na rua (que não sejam policiais ou pessoas autorizadas a usar armas de fogo) |
| People using alcoholic drink on the street | Pessoas usando bebida alcoólica na rua |
| Someone was assaulted with a gun or knife | Alguém foi assaltado com uma arma de fogo ou faca |
| Someone has suffered some form of sexual violence. | Alguém sofreu algum tipo de violência sexual. |
| Someone was assaulted by a police officer. | Alguém foi agredido por um policial. |
| Someone bribed a police officer. | Alguém subornou um policial. |

**Positive parenting**

| You and your parents - Please, mark with crosses which of the things below occur never, rarely, sometimes or often in your home. "Parents" are the adults that care for you at home.  Never / rarely / sometimes / Often/always | Você e seus pais - Por favor, marque se o que está escrito nas frases abaixo nunca, raramente, às vezes ou frequentemente acontecem na sua casa. Aqui o termo "pais" refere-se aos adultos que cuidam de você em csa.  Nunca / Raramente / Às vezes / Frequentemente |
| --- | --- |
| Your parents let you know when you have done a good job with something. | Quando você faz algo bem feito, seus pais reconhecem. |
| Your parents reward you when you have done a good job with something. | Quando você faz bem alguma coisa, seus pais o recompensam, dão um prêmio ou presente a você. |
| Your parents compliment you if you were particularly good at school, in a sport, or at a hobby. | Seus pais elogiam você se você se sair muito bem na escola, no esporte ou alguma atividade de lazer. |

**Peer delinquency**

| Do people in your group do illegal things together? (eg taking drugs, robbing stores or destroying things)  Yes No | As pessoas do seu grupo fazem juntas coisas ilegais? (p.ex. consumir drogas, roubar lojas ou destruir coisas)  Sim / Não |
| --- | --- |

**Socio-economic status**

| Do you have your own cell phone or smartphone? | Você tem celular próprio? |
| --- | --- |
| Do you have your own computer with internet access in your room? | Você tem computador com internet no seu quarto? |
| Do you have your own television in your room (including watching TV over the internet)? | Você tem uma televisão no seu quarto (inclui assistir televisão pela internet no quarto)? |
| Do you have a landline (conventional telephone) in your house?* | Na sua casa tem telefone fixo (convencional)? |
| Does anyone who lives in your house have a car?* | Alguém que mora na sua casa tem carro?* |
| Is there a domestic employee (housekeeper) receiving money to do the work in your home, five or more days a week? * | Tem empregado(a) doméstico(a) recebendo dinheiro para fazer o trabalho em sua casa, cinco ou mais dias por semana? * |
| How many bathrooms with shower are there inside your house?* | Quantos banheiros com chuveiro tem dentro da sua casa? * |

*Additional items of the Brazilian instrument.
